# The 3′UTR signature defines a highly metastatic subgroup of triple-negative breast cancer

**DOI:** 10.18632/oncotarget.10975

**Published:** 2016-08-01

**Authors:** Lei Wang, Xin Hu, Peng Wang, Zhi-Ming Shao

**Affiliations:** ^1^ Department of Breast Surgery, Fudan University Shanghai Cancer Center, Fudan University, Shanghai, China; ^2^ Key Laboratory of Breast Cancer in Shanghai, Fudan University Shanghai Cancer Center, Fudan University, Shanghai, China; ^3^ Department of Oncology, Shanghai Medical College, Fudan University, Shanghai, China; ^4^ Key Laboratory of Systems Biology, Shanghai Advanced Research Institute, Chinese Academy of Sciences, Shanghai, China; ^5^ School of Life Science and Technology, ShanghaiTech University, Shanghai, China; ^6^ Institutes of Biomedical Sciences, Fudan University, Shanghai, China

**Keywords:** prognostic modeling, 3′ untranslated region, alternative polyadenylation, triple-negative breast cancer, biomarker

## Abstract

Triple-negative breast cancer (TNBC) is a highly heterogeneous disease with an aggressive clinical course. Prognostic models are needed to chart potential patient outcomes. To address this, we used alternative 3′UTR patterns to improve postoperative risk stratification. We collected 327 publicly available microarrays and generated the 3′UTR landscape based on expression ratios of alternative 3′UTR. After initial feature filtering, we built a 17-3′UTR-based classifier using an elastic net model. Time-dependent ROC comparisons and Kaplan–Meier analyses confirmed an outstanding discriminating power of our prognostic model for TNBC patients. In the training cohort, 5-year event-free survival (EFS) was 78.6% (95% CI 71.2–86.0) for the low-risk group, and 16.3% (95% CI 2.3–30.4) for the high-risk group (log-rank *p*<0.0001; hazard ratio [HR] 8.29, 95% CI 4.78–14.4), In the validation set, 5-year EFS was 75.6% (95% CI 68.0–83.2) for the low-risk group, and 33.2% (95% CI 17.1–49.3) for the high-risk group (log-rank *p*<0.0001; HR 3.17, 95% CI 1.66–5.42). In conclusion, the 17-3′UTR-based classifier provides a superior prognostic performance for estimating disease recurrence and metastasis in TNBC patients and it may permit personalized management strategies.

## INTRODUCTION

Triple-negative breast cancer (TNBC) which lacks estrogen receptor (ER) and progesterone receptor (PR) expression and without human epidermal growth factor receptor 2 (HER2) amplification, accounts for 15–20% of breast cancers [1]. It is a highly heterogeneous disease with an aggressive clinical course. Survival dramatically decreases during the first 3 to 5 years after diagnosis and distant relapse after this period is rare, indicating a potential curability of TNBC.

Treatment selection should be tailored to the patient based on the risk of recurrence and it should include considerations of tumor size, lymph node and receptor status and patient age at diagnosis [2]. In the era of personalized medicine, traditional clinicopathological risk factors are being challenged [3] and novel biomarkers and multigene expression assays [4–6] are emerging as better predictors of patient outcomes. However, few risk prediction models are widely used in clinical practice so the need remains to distinguish TNBC patients with poor prognosis from those with potentially favorable outcomes, and to test alternative treatment modalities with higher toxicity for these high-risk patients.

Alternative polyadenylation (APA) is a fundamental molecular mechanism influencing the kinetics of gene regulation under diverse physiological and pathological states through the dynamic usage of messenger RNA (mRNA) 3′ untranslated regions (3′UTRs) [7, 8]. Virtually 70% of human genes have multiple polyA sites that produce distinct transcripts with variable 3′UTR length and this contributes to transcriptome diversity [9, 10]. Recent studies [11–13] suggest a biological importance of APA dynamics in human cancer, but the precise role in carcinogenesis is pooly defined. We previously demonstrated the prognostic relevance of 3′UTR APA dynamics in prostate cancer. Consensus clustering with 3′UTR APA data stratified patients with prostate cancer into four subsets with different risk of biochemical relapse [14], indicating that a 3′UTR signature can be used to stratify risk in cancer. For breast cancer, only a few studies describe the 3′UTR landscape in clinical specimens and most focus exclusively on the prognostic significance of 3′UTR shortening, regardless of the heterogeneity between different molecular subtypes [15, 16]. Data are still scarce for APA dynamics of TNBC and its survival relevance, so we estimated the underlying prognostic value of a 3′UTR signature in TNBC including both 3′UTR shortening and lengthening. Then we developed a multi-3′UTR-based model with an elastic net model to identify a subgroup of operable TNBC with high metastatic potential. We evaluated the prognostic efficacy of this classifier in training and validation sets and compared it to traditional clinical risk factor assessment and single 3′UTRs. To our knowledge, this is the first study that illustrates the prognostic significance of a 3′UTR signature in TNBC.

## RESULTS

### Patient characteristics

Patients (*n* = 327) with TNBC were retrospectively recruited to this study. Patients were on average 52.5 years-of-age (SD 12.4 years; median 53 years, IQR 43–61, range 29–84). After a median follow-up time of 88 months, 114 of the 327 patients had tumor relapse or distant metastasis.

For robust prognostic modeling, we divided TNBC data into training (*n* = 164) and validation sets (*n* = 163) using stratified random sampling based on microarray batches. Baseline clinicopathological characteristics are summarized in Table 1. There was no statistically significant difference in the event-free survival time between TNBC patient sets (log-rank *p* = 0.89) or age (mean 52.4 years-of-age; IQR 43–61 for training set; mean 52.6 years-of-age; IQR 43–61 for validation set *p* = 0.465). Lymph node status and tumor size distributions did not vary between patients in either set (*p* = 0.977 and 0.974, respectively).

**Table 1:** Clinical characteristics of patients by 3′UTR assessment set

	Training set				Validation set			
	Number of patients	Low risk(%)	High risk(%)	*p-*value	Number of patients	Low risk(%)	High risk(%)	*p-*value
**Age**				0.88				0.84
>50 years	84	69 (82.1)	15 (17.9)		86	67 (77.9)	19 (22.1)	
≤50 years	80	65 (81.3)	15 (18.8)		77	61 (79.2)	16 (20.8)	
**Lymph node status**				0.70				0.21
negative	130	107 (82.3)	23 (17.7)		129	104 (80.6)	25 (19.4)	
positive	34	27 (79.4)	7 (20.6)		34	24 (70.6)	10 (29.4)	
**Tumor size**				0.48				0.43
≤2 cm	41	35 (85.4)	6 (14.6)		41	34 (82.9)	7 (17.1)	
>2 cm	123	99 (80.5)	24 (19.5)		122	94 (77.0)	28 (23.0)	

We generated 3′UTR ERI profiles of 1,933 genes from 12 microarray batches and removed batch effects in ERI data using the ComBat method, which incorporates systematic batch biases common across variables for adjustments. Supplementary Figure S1 shows thatsamples mixed well in the principle component analysis (PCA), indicating corrected batch effects.

### A 17-3′UTR-based classifier redefines patients with different risks of relapse or metastasis

After applying initial feature filtering using univariate Cox analysis, we developed a prognostic model with an elastic net to the training TNBC samples set. A value λ = 0.109 with log(λ) = −2.22 was chosen by leave-one-out cross validation via 1-SE criteria. The optimal penalty parameter selected 17 3′UTRs in the elastic net model and risk scores were calculated for all patients as a weighted sum of selected features based on this model. The ultimate model is a linear combination of 3′UTRs (features) selected by the elastic net. The weights are an estimation of the information contributed by each marker. Specifically:

(Equation 1) $$\begin{array}{l} \text{Risk score}=-0.104\times SMAD6-0.125\times CXCL8-0.174\\ \times CLIC2-0.213\times PRCKB-0.232\times RTN1-0.292\times \\ ZCCHC14-0.292\times PPIC+0.090\times SIK3+0.119\\ \times UBE2G2+0.175\times SCL2A3+0.188\times \\ SYNGR1+0.213\times NPL+0.271\times PRSS12+0.304\\ \times ADGRL2+0.364\times ZER1+0.453\times WDHD1+0.527\times N4BP2L2 \end{array}$$

The exponential of the coefficient gives the hazard ratio (HR) of event (recurrent or metastasis) associated with each marker. Intuitively, we computed a standardized risk score and parameters of linear transformation were determined by the training set. Specifically:

(Equation 2) $$\text{standardized risk score}=\frac{\text{risk score}-0.625}{0.578}$$

Supplementary Figure S2 shows that standardized risk score distribution was unimodal with similar peaks between training and validation cohorts. To select a threshold to stratify TNBC into high- and low-risk groups, we use X-tile approach, which split training patients into two risk groups with maximal χ^2^ log-rank value (Supplementary Figure S3). We included patients with risk scores > 1.146 (standardized risk score 0.903) as high risk of recurrence or distant metastasis (high-risk group), and those with risk score < 1.146 as low risk of recurrence or metastasis (low-risk group).

**Figure 1:**
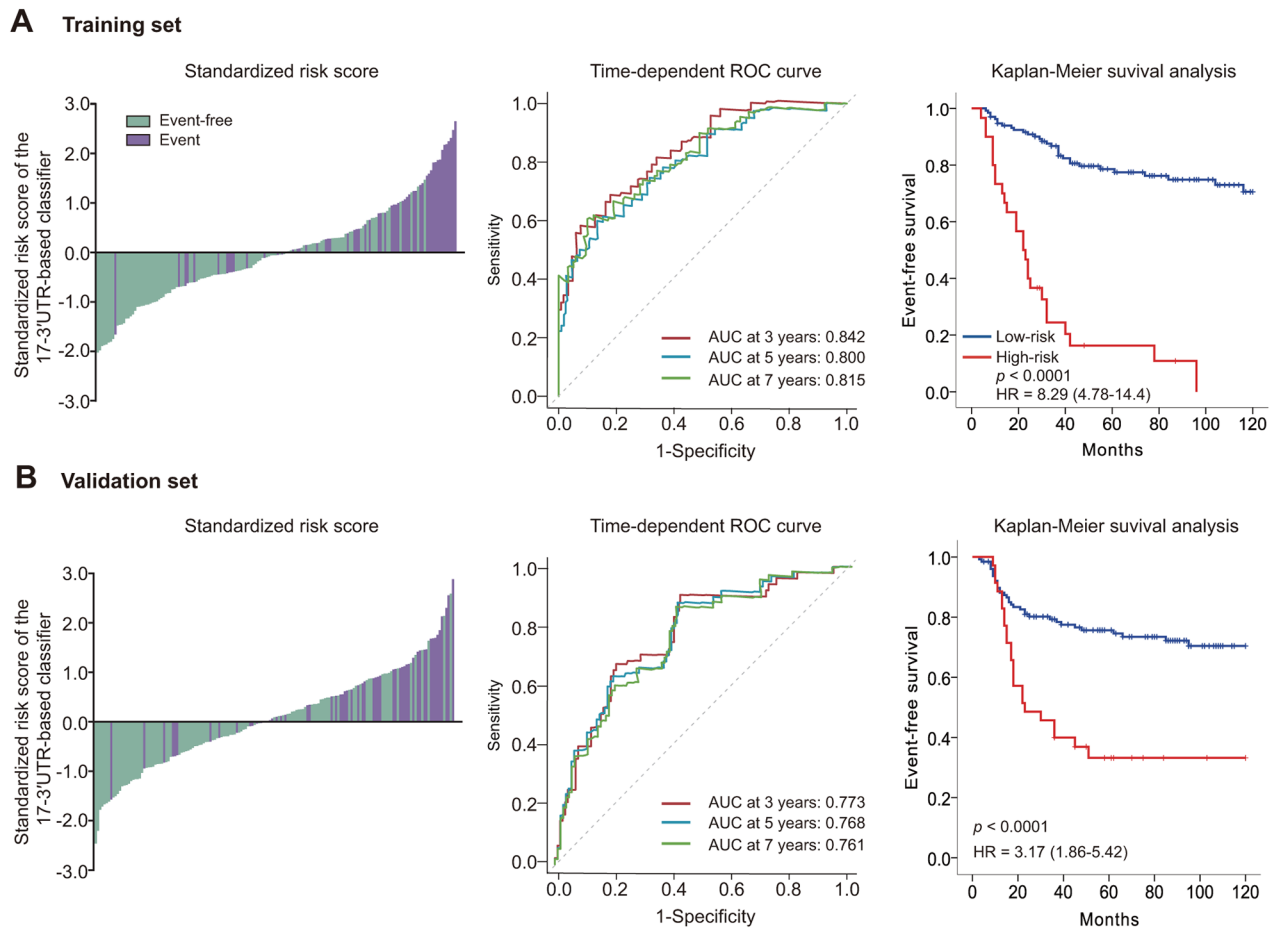
Risk score by the 17-3′UTR-based classifier, time-dependent ROC curves and Kaplan–Meier survival curves in the training and validation sets Data are bootstrap-corrected AUC or hazard ratio (95% CI). ROC=receiver operator characteristic. AUC=area under the curve. **A.** Training set. **B.** Validation set.

In the training set, high-risk patient prognosis was much worse (log-rank *p*<0.0001; hazard ratio [HR] 8.29, 95% CI 4.78–14.4). Also, 5-year EFS was 16.3% (95% CI 2.3–30.4) for the high-risk group, and 78.6% (95% CI 71.2–86.0) for the low risk group (Figure 1A). The prognostic accuracy of the 17-3′UTR-based model to distinguish recurrent and non-recurrent TNBCs was assessed using time-dependent ROC curves. Bootstrap-corrected area under the curve (AUC) at 3, 5, and 7 years were 0.842 (95% CI 0.771–0.912), 0.800 (95% CI 0.718–0.882) and 0.815 (95% CI 0.738–0.892), respectively (Figure 1A).

To assess the robustness of the 17-3′UTR-based model, we applied it to validation set samples using the same threshold and divided validation set patients into two risk groups. Survival analysis revealed a significant difference between high- and low-risk groups (log-rank *p* < 0.0001; HR 3.17, 95% CI 1.66–5.42) and the 5-year EFS was 33.2% (95% CI 17.1–49.3) for the high-risk group, and 75.6% (95% CI 68.0–83.2) for the low-risk group (Figure 1B). Bootstrap-corrected AUC at 3, 5, and 7 years were 0.773 (95% CI 0.692–0.855), 0.768 (95% CI 0.689–0.846) and 0.761 (0.680–0.841), respectively (Figure 1B).

**Figure 2:**
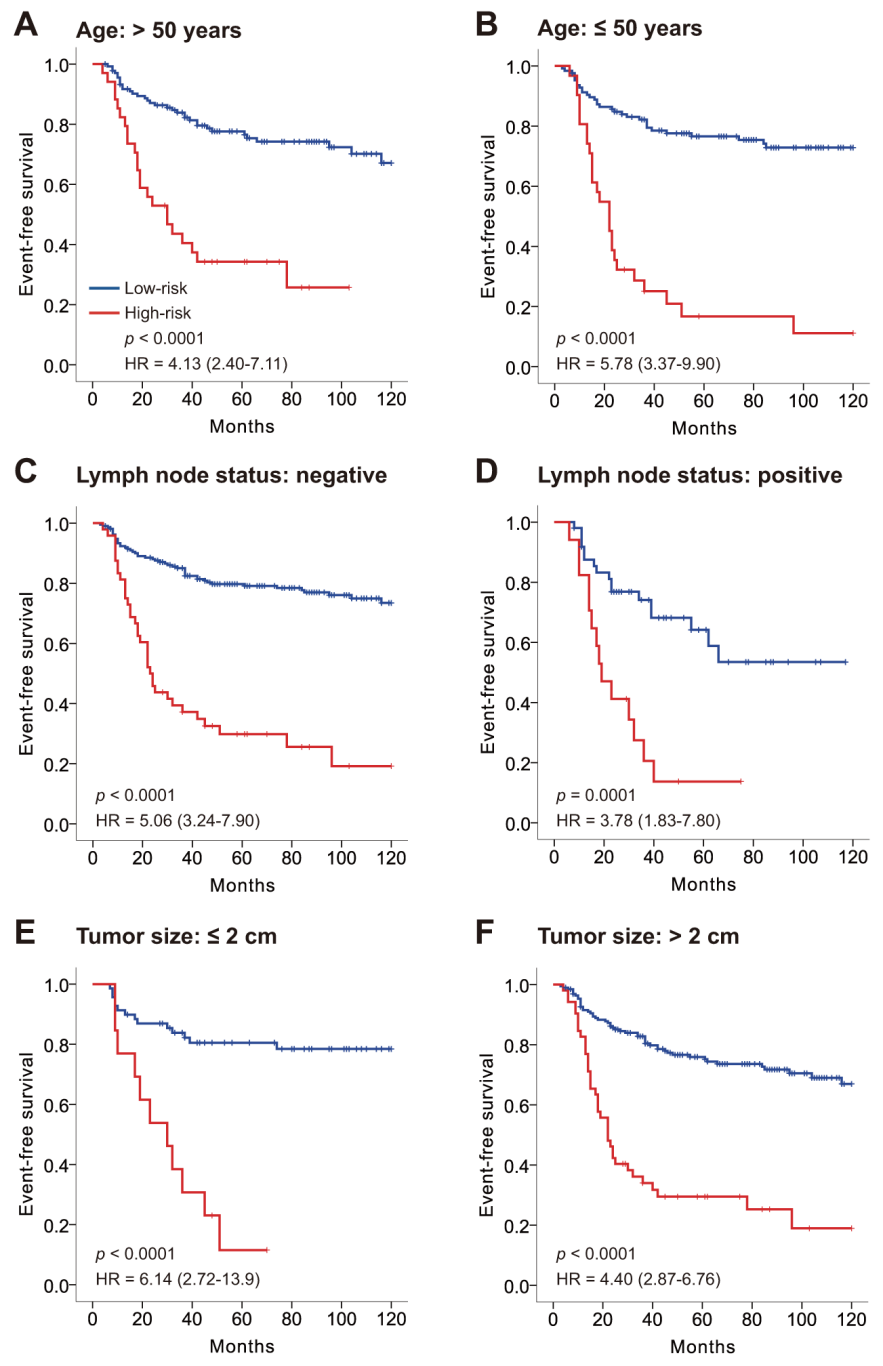
Kaplan–Meier survival analysis for all 327 patients with triple negative breast cancer according to the 17-3′UTR-based classifier stratified by clinicopathological risk factors **A, B.** Age. **C, D.** Lymph node status. **E, F.** Tumor size. *P*-values were calculated using a log-rank test.

We then investigated the relationship between the 17-3′UTR-based classifier and available clinical risk factors (age, lymph node status and tumor size). Table 1 confirms no significant correlation between the risk group and clinical factors in both sets (*p* > 0.05 for all). Stratified by age, lymph node status and tumor size, the 17-3′UTR-based classifier could still differentiate patients with high- and low-risk TNBC among all subgroups (Figure 2).

### 17-3′UTR-based classifier adds significant prognostic information to established clinicopathologic risk factors

We evaluated the additional prognostic power of the 17-3′UTR-based classifier compared to clinical variables with using a univariate and multivariate Cox proportional hazards model. We included three clinical risk factors (age at diagnosis, lymph node status and tumor size) that were available for all patients in our analysis. The 17-3′UTR-based classifier and lymph node status were consistently significant across the different data sets (*p*<0.05; Table 2). After multivariate adjustment by clinicopathological variables, the 17-3′UTR-based classifier retained significant prognostic power in all 327 patients. (HR 4.72, 95% CI 3.22–6.92; *p*<0.0001; Supplementary Table 1). The multivariate Cox analysis identified two independent prognostic factors for EFS: the 17-3′UTR-based classifier was the strongest predictor, followed by lymph node status.

**Table 2:** Univariate association of 17-3′UTR-classifier, clinicopathological variables, and single 3′UTR ERI with event-free survival

	Training set (*n*=164)		Validation set (*n*=163)	
	HR (95% CI)	*p*-value	HR (95% CI)	*p*-value
Age (>50 years *vs* ≤50 years)	1.17 (0.69-1.96)	0.56	1.01 (0.60-1.70)	0.96
Lymph node status (negative *vs* positive)	1.95 (1.06-3.60)	0.032	1.80 (1.02-3.19)	0.044
Tumor size (≤2 cm *vs* >2 cm)	1.10 (0.60-2.01)	0.76	1.51 (0.78-2.91)	0.22
*CLIC2* 3′UTR	0.52 (0.28-0.96)	0.036	0.61 (0.36-1.03)	0.063
*CXCL8* 3′UTR	0.61 (0.45-0.84)	0.0021	0.72 (0.54-0.97)	0.032
*PPIC* 3′UTR	0.32 (0.15-0.68)	0.0030	0.55 (0.25-1.23)	0.15
*PRCKB* 3′UTR	0.47 (0.27-0.80)	0.0055	0.60 (0.34-1.06)	0.077
*RTN1* 3′UTR	0.38 (0.16-0.91)	0.030	0.39 (0.17-0.86)	0.019
*SMAD6* 3′UTR	0.48 (0.29-0.81)	0.0062	0.72 (0.47-1.09)	0.12
*ZCCHC14* 3′UTR	0.55 (0.33-0.91)	0.021	0.70 (0.43-1.13)	0.15
*ADGRL2* 3′UTR	3.04 (1.34-6.91)	0.0080	2.37 (1.09-5.14)	0.030
*N4BP2L2* 3′UTR	2.77 (1.52-5.05)	0.00092	1.70 (0.91-3.18)	0.099
*NPL* 3′UTR	1.80 (1.00-3.22)	0.048	1.70 (0.98-2.96)	0.061
*PRSS12* 3′UTR	1.89 (0.80-4.49)	0.15	2.27 (1.20-4.30)	0.012
*SCL2A3* 3′UTR	2.20 (1.31-3.67)	0.0027	1.61 (0.96-2.68)	0.068
*SIK3* 3′UTR	2.34 (1.18-4.67)	0.015	1.92 (0.94-3.94)	0.073
*SYNGR1* 3′UTR	1.72 (0.93-3.18)	0.084	2.36 (1.26-4.42)	0.0072
*UBE2G2* 3′UTR	1.73 (0.98-3.04)	0.057	1.63 (1.01-2.64)	0.046
*WDHD1* 3′UTR	7.56 (2.01-28.6)	0.0028	3.01 (0.70-12.9)	0.14
*ZER1* 3′UTR	3.20 (1.32-7.75)	0.0099	1.76 (0.84-3.70)	0.13
17-3′UTR-based classifier	8.73 (5.05-15.1)	<0.0001	3.22 (2.10-4.94)	<0.0001
17-3′UTR-based classifier (low *vs* high risk)	8.29 (4.78-14.4)	<0.0001	3.17 (1.86-5.42)	<0.0001

**Figure 3:**
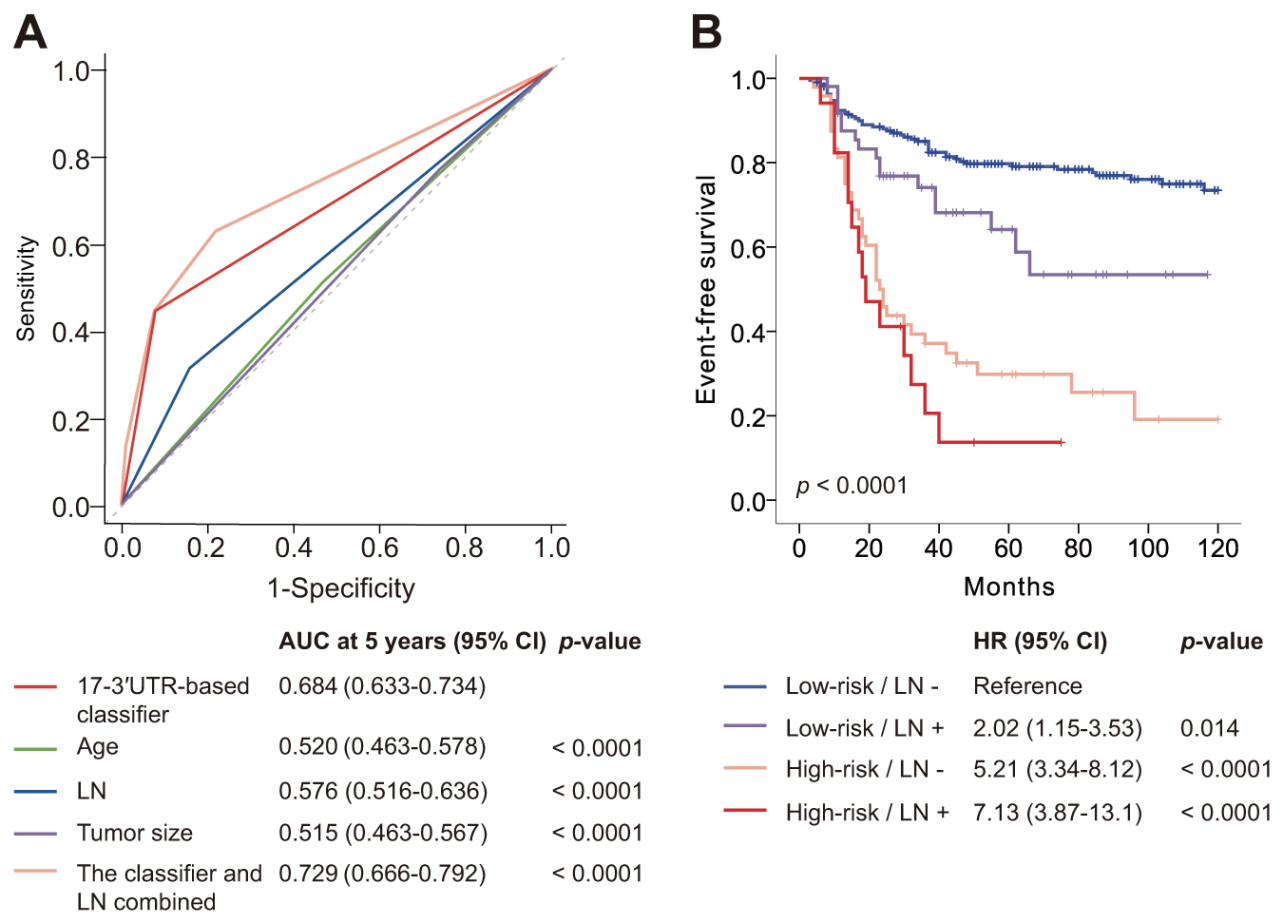
Time-dependent ROC curves compare the prognostic power of the 17-3′UTR-based classifier with clinicopathological risk factors, and Kaplan–Meier survival analysis for patients stratified by the classifier and lymph node status ROC=receiver operator characteristic. AUC=area under the curve. **A.** Comparisons for the prognostic accuracy by the 17-3′UTR-based classifier (high risk *vs* low risk), age (≤50 years *vs* >50 years), lymph node status (positive *vs* negative), tumor size (≤2 cm *vs* >2 cm), or the classifier and lymph node status combined. **B.** Kaplan–Meier survival analysis shows significant difference among the four groups: low-risk/LN- (*n* = 211), low-risk/LN+ (*n* = 51), high-risk/LN- (*n* = 48), high-risk/LN+ (*n* = 17).

Next, we compared the prognostic accuracy of the classifier and clinical variables. The 17-3′UTR-based classifier had significantly more prognostic power compared to clinicopathological risk factors (*p*<0.0001 for all; Figure 3A). Combining the classifier and lymph node status increased the AUC at 5 years from 0.684 (95% CI 0.633–0.734) to 0.729 (95% CI 0.666–0.792; *p*<0.0001; Figure 3A), indicating that joint use both indices improved the TNBC patient risk prediction.

Using a combination of the 17-3′UTR-based classifier and lymph node status, all patients were classified into four subgroups: low-risk/LN- (*n* = 211), low-risk/LN+ (*n* = 51), high-risk/LN- (*n* = 48), high-risk/LN+ (*n* = 17). Figure 3B shows survival curves of all four groups. Differences in EFS were significant among the four groups (log-rank *p* < 0.0001). Patients with no positive lymph node and low-risk had a low risk of relapse and metastasis, with a 5-year EFS of 79.7% (95% CI 85.2–74.2), whereas the 5-year EFS for patients with positive lymph node status and high-risk was 13.7% (95% CI -3.7–31.1).

### Association between 3′UTR APA dynamics and event-free survival in TNBC

**Figure 4:**
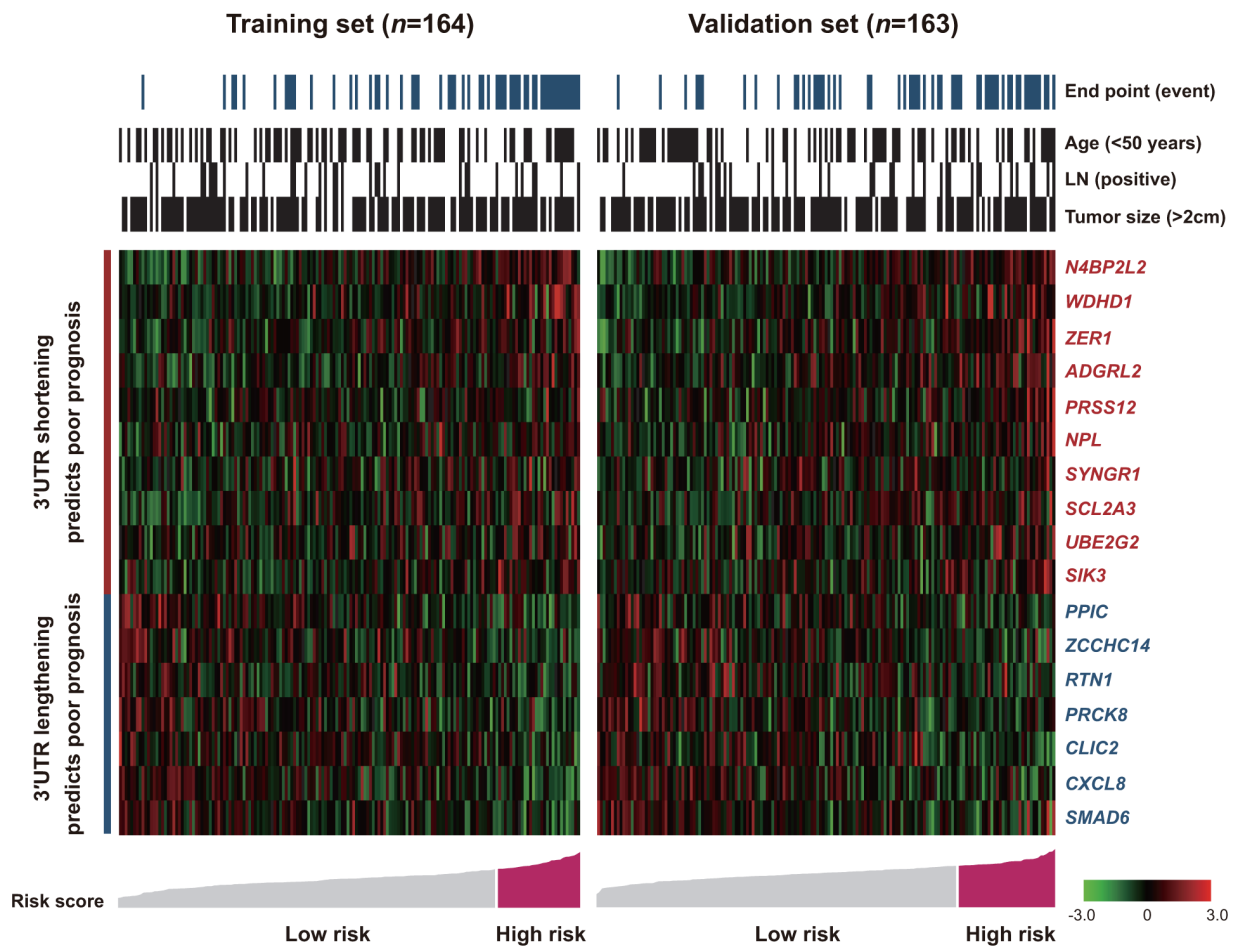
Shortening and lengthening patterns for the 3′UTR markers, patient outcomes and risk scores Risk groups were defined by the risk score with the most significant (log-rank test) split of the training samples.

Figure 4 depicts the distribution of 17 3′UTR ERI levels, patient risk score, clinicopathological factors, and the EFS status of the training and validation sets. Two distinct groups of 3′UTR APA dynamics emerged in regard to prognosis: 3′UTR shortening and 3′UTR lengthening. The association between 3′UTR APA dynamics and EFS in patients with TNBC was evaluated by Kaplan–Meier and Cox proportional hazard regression analyses (Supplementary Figure S4, S5 and Table 2).

Patients in the training set were divided into two groups based on 3′UTR ERIs. The optimal threshold for dichotomizing ERI to plot survival curves was derived from the training cohort using the X-tile program and was applied without modification to the validation set. 3′UTRs with ERIs exceeding the threshold were “shortened” and those at the threshold or less were “lengthened”. These parameters were also applied to the validation set. In 10 of 17 selected 3′UTRs (*N4BP2L2*, *WDHD1*, *ZER1*, *ADGRL2*, *PRSS12*, *NPL*, *SYNGR1*, *SCL2A3*, *UBE2G2* and *SIK3*), 3′UTR shortening was correlated with poorer prognosis (Supplementary Figure S4; Table 2). In the remaining seven 3′UTRs (*PPIC*, *ZCCHC14*, *RTN1*, *PRCK8*, *CLIC2*, *CXCL8* and *SMAD6*), 3′UTR lengthening offered an unfavorable prognosis (Supplementary Figure S5; Table 2).

We also assessed the prognostic accuracy of single 3′UTR and the 17 3′UTR signature to distinguish highly metastatic TNBC using a time-dependent ROC test (Supplementary Figure S6). When considered individually, the 17 3′UTRs had moderate bootstrap-corrected AUCs at 5 years (from 0.528 to 0.605). The 17-3′UTR-based classifier had greater AUC than that of any single 3′UTR (*p*<0.0001 for all).

## DISCUSSION

Increasing recognition of the active role of 3′UTR APA dynamics in tumorigenesis has led to the identification of novel APA markers for prognosis. We developed and validated a novel prognostic model based on 17 3′UTRs to improve the prediction of disease recurrence or distant metastasis for patients with operable TNBC. Data show that the proposed classifier can successfully identify a patient subgroup with poorer 5-year EFS. In addition, this tool can reliably predict risk in patients with TNBC significantly better than the classical clinicopathological risk factors (age at diagnosis, lymph node status, and tumor size). When patients were stratified by risk factors, the 17-3′UTR-based classifier retained prognostic efficacy and offered additional power that complemented clinicopathological factor analysis. To our knowledge, this is the first study to report using the 3′UTR signature in TNBC for patient prognosis.

In the present study, we confirmed that shortening of ten 3′UTRs (*N4BP2L2*, *WDHD1*, *ZER1*, *ADGRL2*, *PRSS12*, *NPL*, *SYNGR1*, *SCL2A3*, *UBE2G2* and *SIK3*) is associated with unfavorable prognosis in TNBC. This is consistent with the emerging role of 3′UTR shortening which enables key genes to escape microRNA repression and cause accelerated tumor progression [11, 12]. Most genes with shortened 3′UTRs in TNBC participate in cancer development. *WDHD1* acts as a cell cycle regulator and a downstream molecule in the PI3K/Akt pathway in lung and esophageal carcinogenesis [17]. NPL glycoprotein serves as a serum biomarker to distinguish healthy and precancerous esophageal lesions [18]. *SYNGR1* has differential expression between bladder cancer with different risk of recurrence [19]. *UBE2G2* is a mutant gene in human leukemia [20] and *SIK3* has been recently identified as a tumor antigen associated with ovarian cancer tumorigenesis. The 3′UTR markers for which lengthening indicates poorer prognosis include *PPIC*, *ZCCHC14*, *RTN1*, *PRCK8*, *CLIC2*, *CXCL8* and *SMAD6*. Among them, *SMAD6* and *CLIC2* are both related to the TGF-β pathway, and *SMAD6* is associated with favorable survival in lung cancer [21, 22]. *CXCL8* participates in the autocrine NF-κB/IL-8 (CXCL8) pathway driving cell migration [23]. Previous studies have mainly focused on the prognostic significance of 3′UTR shortening, but our findings first propose that 3′UTR lengthening can reduce survival. We speculate that more microRNA response elements (MREs) are harbored in the lengthened 3′UTR, leading homologous gene repression and competing endogenous RNA (ceRNA), which causes a series of aberrant pathways and cancer progression. Using computational network analysis, our recent study [14] revealed alternative 3′UTR participates in ceRNA network dynamics in cancer but detailed mechanisms remain to be characterized.

Previous studies identified multiple 3′UTRs that with different length preferences in TNBC compared with non-TNBC or normal breast tissue [13, 24]. Two studies independently confirmed that 3′UTR shortening predicts unfavorable outcomes in breast cancer based on public high-throughput data obtained from GEO and The Cancer Genome Atlas (TCGA), respectively [15, 16]. However, research in cell lines indicated that TNBC prefers longer forms of 3′UTR compared with luminal subtype tissue and normal breast tissue [13]. Previous studies are limited by small numbers of 3′UTR analyzed, small sample sizes and a lack of validation. Although this preliminary work laid the groundwork for exploring the importance of 3′UTR shortening as a promising marker in cancer, our findings are more broad and inclusive of 3′UTR dynamics in human malignancy. With a high-dimensional elastic net modeling technique, we established a 3′UTR signature (including 3′UTR shortening and lengthening) to be a powerful tool for TNBC patient risk stratification by correlating alternative 3′UTR patterns with survival outcomes.

The proposed classifier is of clinical importance because current gene expression prognostic models such as the 70-gene signature [4], the Genomic Grading Index [25], and the Recurrence Score [26] allocate unfavorable prognostic risk category to all TNBC patients despite the diversity of clinical outcomes. Because TNBC is a highly heterogeneous disease, treatment strategies should be personalized according to risk recurrence and our data allow this type of risk evaluation and better management of TNBC patients.

Of note, the statistical method used for analyzing microarray data must be carefully selected. Cox regression was used previously [15] when constructing a prognostic model for breast cancer but this is inappropriate due to an insufficient sample size relative to the high-dimension features (i.e. sample size *n*: feature size *p* less than 10:1), especially for high-dimensional microarray data. An elastic net overcomes this disadvantage (*p* >> *n*) and simultaneously conducts automatic variable and group selections of correlated features [27, 28].

The primary limitation of this study is that it is a retrospective study. Thus, data must be validated using a prospective study in a multicenter clinical trial. In addition, 3′-based chips (HG-U133A and HG-U133 plus 2.0) severely limited the number of genes analyzed (1,933 genes in this study, or ~9.7% of human protein-coding genes). We chose this platform because these two chips are widely used in gene expression studies and the number of available microarray data with survival and clinical information was sufficient for prognostic modeling. We show that transcripts with significant 3′UTR length change are highly biologically and clinically relevant to cancer. However, we cannot ensure our results are unbiased estimations of whole genome profiles among TNBC, so generalizations to patterns of alternative 3′UTR should be performed with caution. Currently we are conducting a study to profile 3′UTR APA dynamics of TNBC using transcriptome arrays, which provide a less biased 3′UTR landscape (paper in preparation).

In summary, we performed the largest 3′UTR APA prognostic analysis of pooled gene expression data and yielded essential clinical information about TNBC. We described a new 3′UTR signature for TNBC that identifies about one-fifth of TNBC patients as being relatively high risk for tumor recurrence. Finally, we studied a limited number of genes, our novel 3′UTR-based classifier should lay a solid foundation for future biological and pathologic analysis of alternative 3′UTR events in TNBC.

## MATERIALS AND METHODS

### TNBC microarray data collection

In this study, we used data from a single Affymetrix platform (including HG-133A and HG-U133 plus 2.0) which is commonly used in microarray studies of breast cancer. Because most microarrays lacked immunohistochemistry information for ER, PR and HER2, triple-negative status was defined based on the bimodal filter of mRNA expression of *ER*, *PR* and *HER2* as previously described [29, 30]. For prognostic efficacy comparisons and stratification analysis, we only included samples with available follow-up data and three established clinicopathological risk factors: age at diagnosis, lymph node status and tumor size. Then, 327 publicly available microarrays were analyzed. Data for 3′UTR analysis comprised 12 pooled public data sets obtained from the Gene Expression Omnibus (GEO), accession GSE31519, GSE29690, GSE2603, GSE2034, GSE5327, GSE11121, GSE7390 and GSE21653.

### 3′UTR profiling and data normalization

Microarrays were analyzed using an R package ‘ERI-expr’ [15], which contains custom chip description files (CDF) for HG-U133A, HG-U133B and HG-U133 plus 2.0. Briefly, the processed probesets matching alternative transcripts with different 3′UTR lengths were extracted in terms of APA sites defined by the polyA_DB database [31]. After robust multi-array average (RMA) correction, intensities for the 5′ (*S*_5′_) and 3′ (*S*_3′_) probe sets included in the custom CDF were extracted. The expression ratio index (ERI) was defined as the signal ratio of 5′ and 3′ probesets of the APA site:

(Equation 3) $$ERI\equiv \frac{S_{5'}}{S_{3'}}=\frac{\alpha_{L}}{\beta_{L}}+\frac{\alpha_{S}e_{S}}{\beta_{L}e_{L}}$$

In equation 3, *e_S_*(*e_L_*) represents expression of the short (long) form of the 3′UTR, whereas α*_S,L_*(β*_S,L_*) denotes the affinities of the short (*S*) or long (*L*) forms for the 5′ (α) and 3′ (β) probe sets. The ERI is a linear function of the expression ration between shortening and lengthening forms of 3′UTR. Thus, we used the ERI value to characterize the relative prevalence of the APA dynamics. With this algorithm, 6,045 APA sites in 3,542 unique genes in HG-U133 plus 2.0 and 3,210 APA sites in 1,933 genes in HG-U133A were identified. Using the intersection of the recognized APA sites between the two chips, we reported the ERI value of the APA site closest to 5′ end of the gene with multiple APA sites, since 90% of the significant changes in APA isoform expression occurred at the first APA site [32].

To minimize batch effects when pooling ERI data, we used an empirical Bayes based ComBat method to adjust batch effects in microarray data [33]. To confirm that batch effects were successfully removed, principle component analysis (PCA) of combined ERI data sets was subsequently performed to visualize the spatial distribution of data sets from different batches.

### Development and validation of risk prediction model for TNBC

3′UTR profiles were obtained from 12 publicly available data sets that contained 327 primary TNBCs. For robust analysis, we used stratified random sampling to assign each sample to a training or validation set according to chip batch. Patient characteristics of training and validation cohorts appear in Table 1. To reduce feature dimensionality, we first filtered out noisy features using univariate Cox analysis applied to 3′UTR ERI data. We established a threshold of 0.15 for the *p-*values, and kept only 3′UTRs with a Wald *p*-value smaller than 0.15 for model development.

Next, we used the elastic net [27] to identify 3′UTRs associated with event-free survival and to train the final model for prognosis with the selected features in the training set. The elastic net has been extended and broadly applied to the Cox proportional hazard regression model for survival analysis with high-dimensional data. This approach conducts automatic feature selection and group selection of the correlated variables simultaneously [28]. To find a parsimonious model with a modest discriminating accuracy, we used ten-fold cross validations to select the penalty parameter λ (the tuning parameter), and chose λ via 1-SE (standard error) criteria. The elastic net mixing parameter α was set to 0.5. We used R version 3.2.3 and its implemented ‘glmnet’ package [34] to perform the elastic net analysis.

To evaluate the effectiveness of the 3′UTR signature for survival prediction, we assigned each patient a risk score according to a linear combination of ERI of the 3′UTRs selected by the elastic net. The risk score function for sample *i* using the information from the significant 3′UTRs was calculated as follows:

(Equation 4) $$\text{risk score}=\sum W_{j}s_{ij}$$

In the above equation, *s_ij_* is ERI for 3′UTR*j* on sample *i*, and *Wj* is the weight of the risk score of 3′UTR*j*. Weights were obtained by the corresponding coefficients derived from the elastic net modeling. The weights are a rough estimate of the information content contributed by each 3′UTR to event-free survival. The standard risk score was also reported for each sample. Then, we allocated the patients to high-risk and low-risk groups using X-tile plots based on correlations with the patients' event-free survival.

X-tile plots adopt an intuitive and concise approach to estimate associations between variables and survival. For continuous variables, X-tile software can analyze each value and choose an optimal threshold with the maximal Chi square value according to the log-rank test (or minimum *p*-value) [35]. We performed X-tile analysis using X-tile version 3.6.1 (Yale University School of Medicine, New Haven, CT).

To confirm the robustness of the elastic net model, we calculated risk scores based on Equation 4 for the validation samples, and divided them into high- or low-risk groups, with the threshold determined in the training set as described above. Time-dependent receiver operating characteristic (ROC) curves, a Cox regression model and Kaplan–Meier survival analysis were used to assess the prognostic accuracy of the model in training and validation sets.

### Statistical analysis

Patient characteristics were summarized for all participants using standard descriptive statistics. A Pearson's *χ*^2^ test was used to compare categorical variables whereas the Student's t-test was used for continuous variables. Disease-free survival (DFS) was preferentially used as a clinical end point for event-free survival (EFS). In data sets without DFS records, distant-metastasis-free survival (DMFS) was used as proxy for DFS. Patients with a study end date or who were lost to follow-up were considered censored. The median follow-up time was calculated using a reverse Kaplan–Meier method and the Kaplan–Meier method was used to construct EFS curves. Log-rank tests were used to assess survival differences. Unadjusted and adjusted hazard ratios (HR) with 95% confidence intervals (CI) were calculated using Cox proportional hazards models.

Time-dependent ROC curves were used to assess the risk prediction model and clinical factors to discriminate among patients with respect to the risk of EFS events [36]. Prediction accuracy was calculated based on the area under the time-dependent ROC curve (AUC). We performed 1,000 bootstrap re-samplings to compute CI for the AUC. In this procedure, rows of data were sampled with replacements, and risk scores and corresponding AUC values were estimated for each iteration.

All reported *p-*values were two sided and *p*<0.05 was considered to be statistically significant. All statistical analyses were completed using SPSS Statistics 20 (SPSS Inc., Chicago, IL) and R 3.2.3 (R Development Core Team, Vienna).

## SUPPLEMENTARY TABLE AND FIGURES


