# Supplementary material for: The 3′UTR signature defines a highly metastatic subgroup of triple-negative breast cancer
Source: Oncotarget. 2016 Aug 1;7(37):59834–44. doi: 10.18632/oncotarget.10975 (PMC5312352; doi:10.18632/oncotarget.10975)
Supplement: Supplementary file 1 [file oncotarget-07-59834-s001.pdf]

The 3′UTR signature defines a highly metastatic subgroup of triple-negative breast cancer

SUPPLEMENTARY TABLE AND FIGURES

Supplementary Table S1: Multivariate Cox regression model for event-free survival

	HR (95% CI)	*p*-value
Age (>50 years *vs* ≤50 years)	1.18 (0.81-1.71)	0.39
LN status (negative *vs* positive)	1.69 (1.10-2.61)	**0.017**
Tumor size (≤2cm *vs* >2cm)	1.04 (0.66-1.64)	0.88
17-3′UTR-based classifier (low-risk *vs* high risk)	4.72 (3.22-6.92)	**<0.0001**

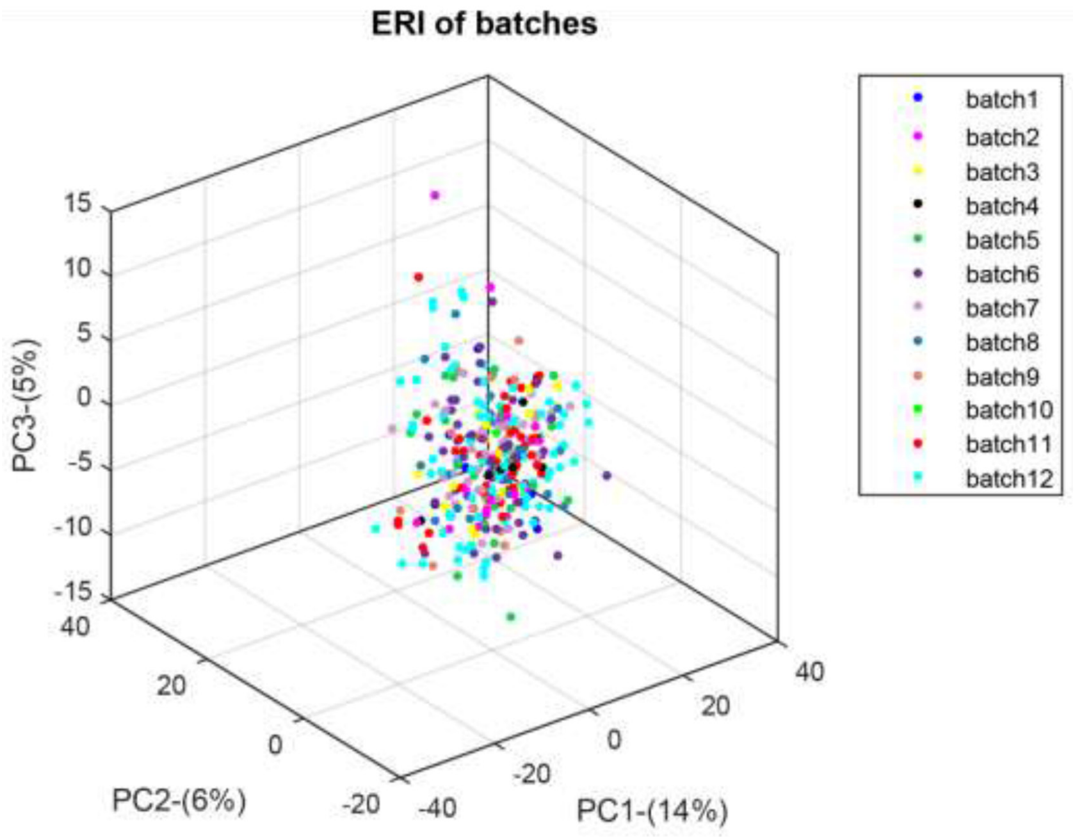

Supplementary Figure S1: Principle component analysis (PCA) of pooled ERI data from 12 batches.

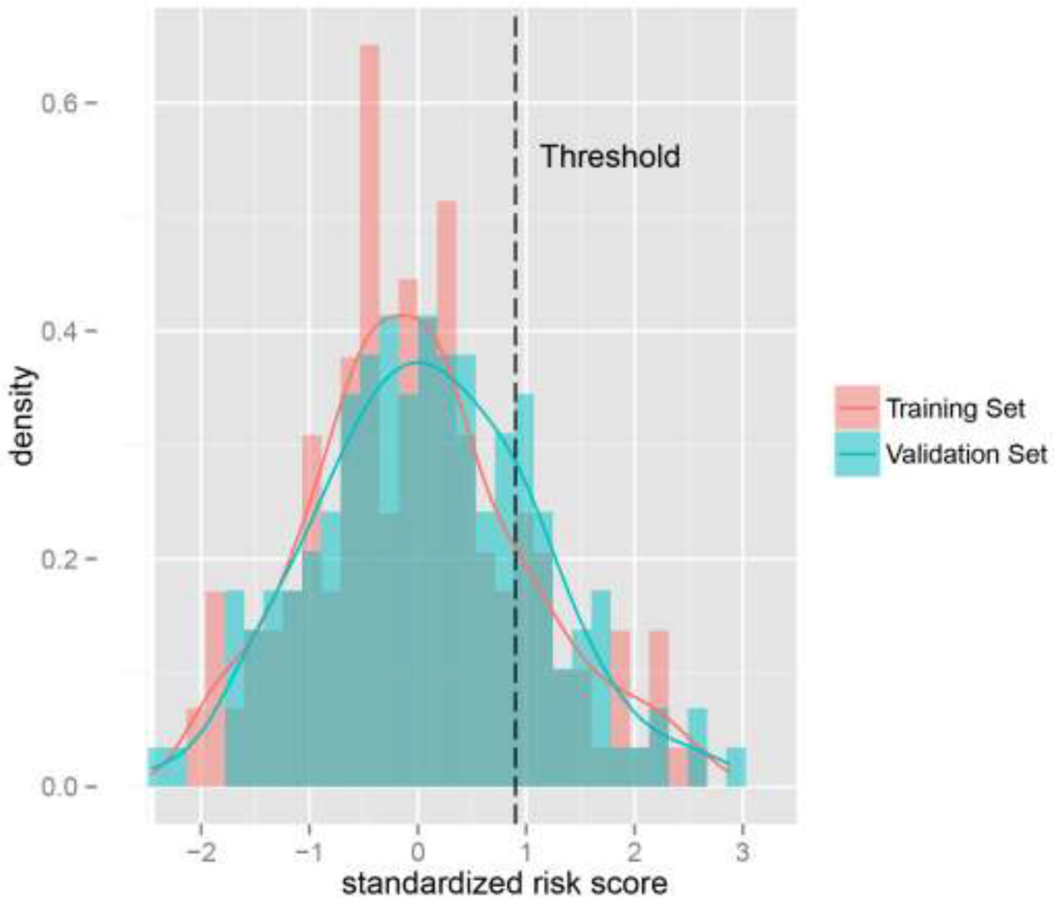

Supplementary Figure S2: The distribution of standardized risk scores of training set (red) and validation set (green). The dashed line denotes the threshold used (0.903).

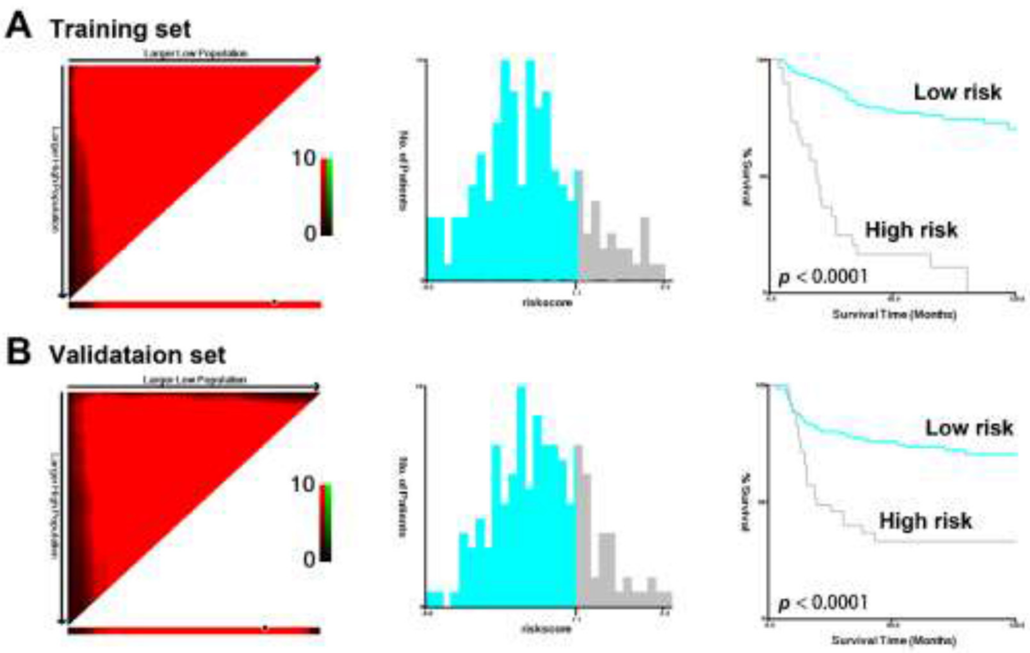

Supplementary Figure S3: X-tile plots of the elastic net risk scores in the training A. and validation B. set. Coloration of the plot represents the strength of the association at each division, ranging from low (dark, black) to high (bright, red or green). Red represents inverse association between risk scores and survival, whereas green represents direct association.

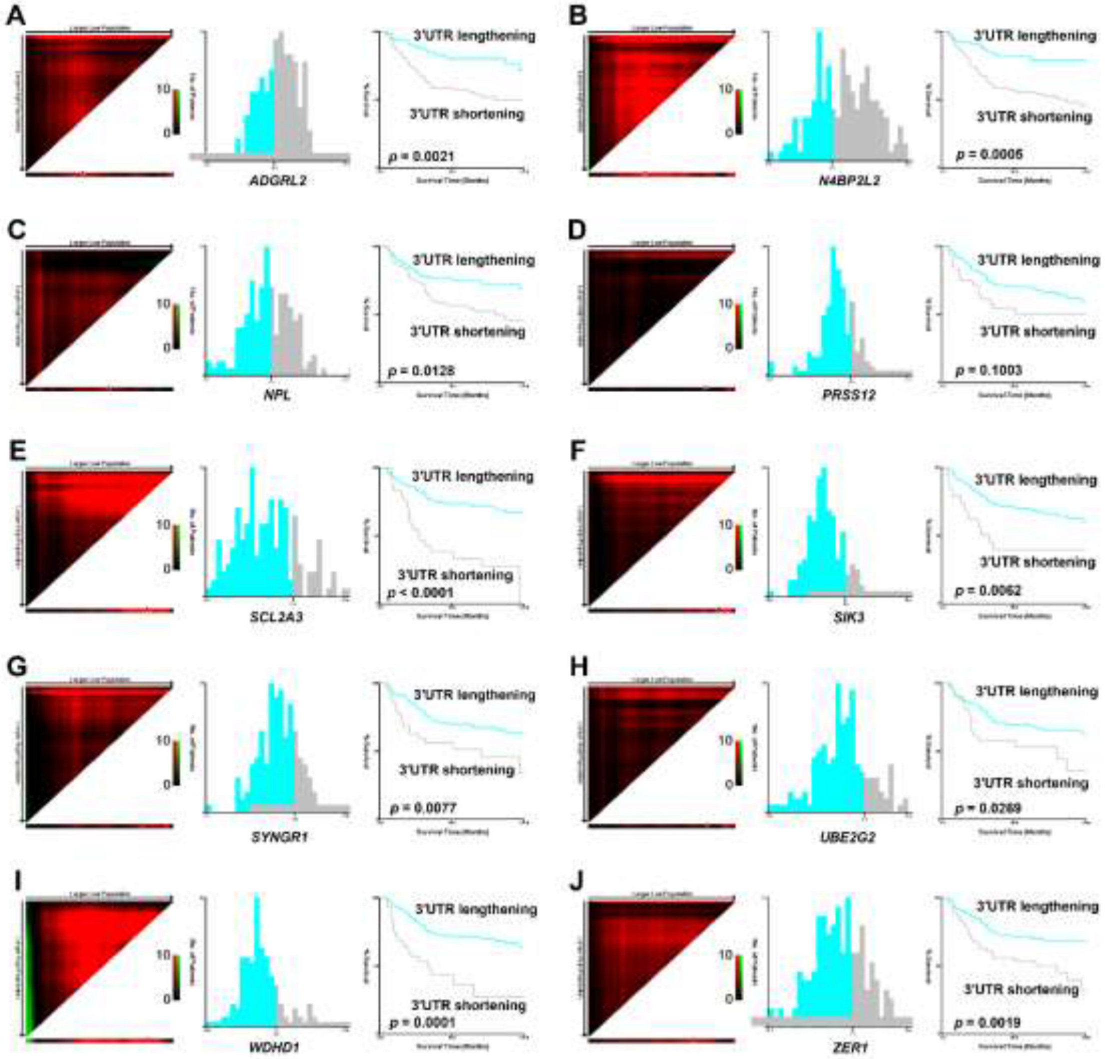

Supplementary Figure S4: X-tile plots of the selected 3′UTRs of which shortening suggests poor prognosis in the training set.

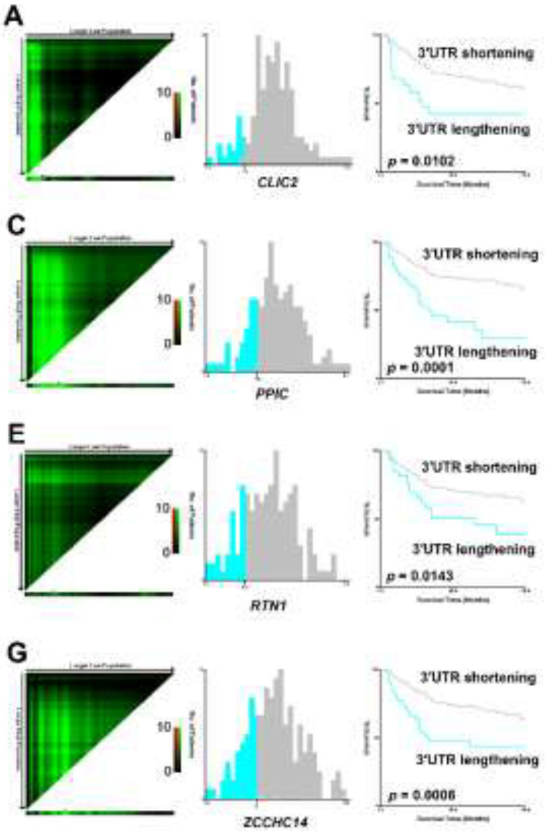

Supplementary Figure S5: X-tile plots of the selected 3′UTRs of which lengthening suggests poor prognosis in the training set.

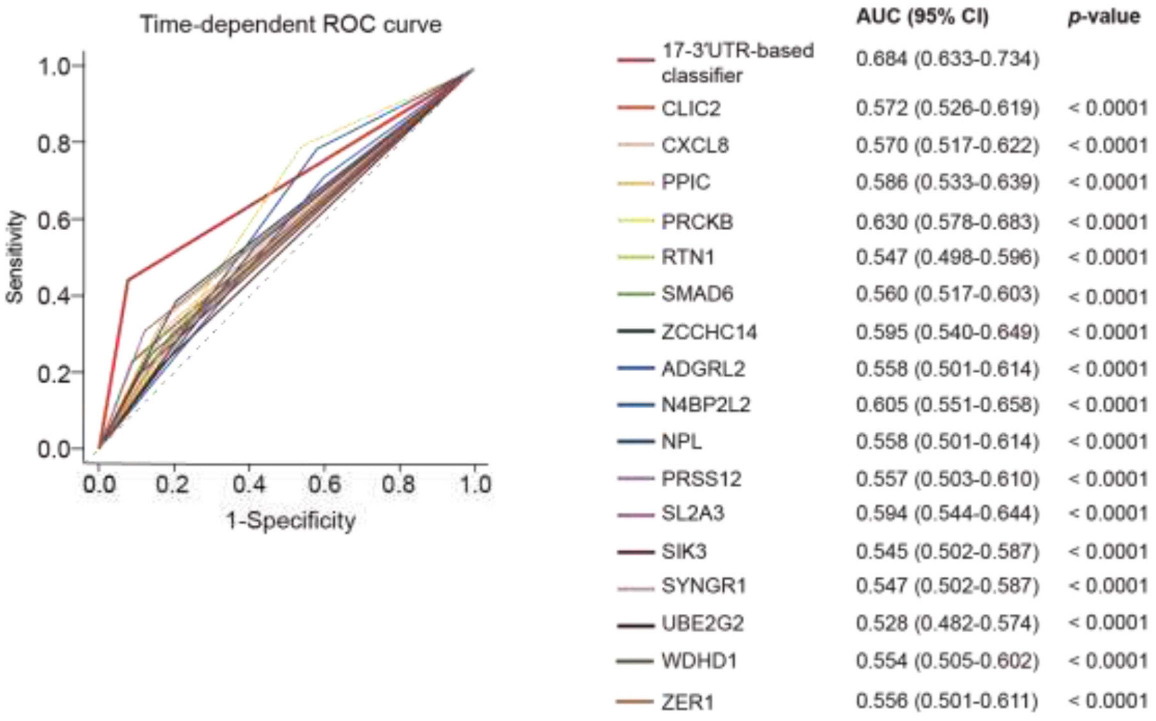

Supplementary Figure S6: Time-dependent ROC curves compare the prognostic accuracy of the 17-3′UTR-based classifier with single 3′UTRs in all 327 patients with TNBC. *P*-value shows the AUC at 5 years for the 17-3′UTR-based classifier *vs* the AUC at 5 year for single 3′UTRs.
